# Revealed willingness-to-pay versus standard cost-effectiveness thresholds: Evidence from the South African HIV Investment Case

**DOI:** 10.1371/journal.pone.0186496

**Published:** 2017-10-26

**Authors:** Gesine Meyer-Rath, Craig van Rensburg, Bruce Larson, Lise Jamieson, Sydney Rosen

**Affiliations:** 1 Department of Global Health, Boston University School of Public Health, Boston, United States of America; 2 Health Economics and Epidemiology Research Office (HE^2^RO), Department of Internal Medicine, School of Clinical Medicine, Faculty of Health Sciences, University of the Witwatersrand, Johannesburg, South Africa; University of New South Wales, AUSTRALIA

## Abstract

**Background:**

The use of cost-effectiveness thresholds based on a country’s income per capita has been criticized for not being relevant to decision making, in particular in middle-income countries such as South Africa. The recent South African HIV Investment Case produced an alternative cost-effectiveness threshold for HIV prevention and treatment interventions based on estimates of life years saved and the country’s committed HIV budget.

**Methods:**

We analysed the optimal mix of HIV interventions over a baseline of the current HIV programme under the committed HIV budget for 2016–2018. We calculated the incremental cost-effectiveness ratio (ICER) as cost per life-year saved (LYS) of 16 HIV prevention and treatment interventions over 20 years (2016–2035). We iteratively evaluated the most cost effective option (defined by an intervention and its coverage) over a rolling baseline to which the more cost effective options had already been added, thereby allowing for diminishing marginal returns to interventions. We constrained the list of interventions to those whose combined cost was affordable under the current HIV budget. Costs are presented from the government perspective, unadjusted for inflation and undiscounted, in 2016 USD.

**Results:**

The current HIV budget of about $1.6 billion per year was sufficient to pay for the expansion of condom availability, medical male circumcision, universal treatment, and infant testing at 6 weeks to maximum coverage levels, while also implementing a social and behavior change mass media campaign with a message geared at increasing testing uptake and reducing the number of sexual partners. The combined ICER of this package of services was $547/ LYS. The ICER of the next intervention that was above the affordability threshold was $872/LYS.

**Conclusions:**

The results of the South African HIV Investment Case point to an HIV cost-effectiveness threshold based on affordability under the current budget of $547–872 per life year saved, a small fraction of the country’s GDP per capita of about $6,000.

## Introduction

Since 2001, an increasing number of economic evaluations of healthcare interventions in low- and middle-income countries have used threshold values to designate healthcare interventions as cost effective. The most commonly used threshold values were, first, gross national income (GNI) per capita per life year saved (LYS), as suggested by the Commission for Macroeconomics and Health [1], and, more recently, 1–3 times gross domestic product (GDP) per capita per disability-adjusted life year (DALY) averted, as suggested by WHO-CHOICE [2]. The indiscriminate use of these thresholds, especially in the absence of consideration for other decision criteria have been criticized repeatedly [3,4], including by WHO-CHOICE [5], but few agreed-upon alternatives exist. Other decision criteria that could be included are whether an intervention or packages of interventions can be provided to all affected populations (equitable coverage), whether it has an impact on alleviating poverty, and whether or not a government or other funder is in fact able to pay for an intervention (affordability). (See Table 1 for explanations of these and other economics concepts used in this paper.)

**Table 1:** Definitions of economics terminology used in the paper.

Affordability	Ability of a funder to pay for an intervention
Coverage	The proportion of the population in need of, or eligible for, an intervention that is receiving it
Disability-adjusted life years	Combined metric of life years lost to a disease and life years lived with less than optimal health (disability)
Earmarked funds	Funding that can only be used for a pre-specified purpose
Equitable coverage	Provision of an intervention to all affected populations and population groups
Fungibility	The interchangeability of individual units of goods or commodities, or funding
Gross domestic product	Total value of goods produced and services provided in a country during a year
Gross national income	Total value of goods produced and services provided by the population of a country during a year (GDP plus net income from investments abroad)
Poverty impact	Effect of intervention on alleviating poverty
Purchasing power	The number and quality of goods and services that can be bought with a unit of a country’s currency
Uniform	Remaining the same over time and in all cases
Willingness-to-pay	Willingness of a funder to pay for an intervention

Utilising an international standard to create a country-specific threshold to assess cost-effectiveness suggests that we know what a society as a whole is willing to pay for a defined unit of health benefit, and that this willingness-to-pay (WTP) is equal, for example, to GDP/capita. Generally, methods for measuring WTP are categorised as stated preference or revealed preference (i.e. based on actual decisions) [6,7]. One way of estimating revealed preferences is to examine the relationship between past expenditure and past survival, for health overall [8] or for specific services [9]. Thresholds based on this method can be thought of as representing the opportunity cost of healthcare spending. Opportunity cost in this context refers to the potential benefit from an investment in health that could have been secured had the resources been invested in the next best alternative. Efforts to generate country-specific estimates of cost-effectiveness thresholds based on health opportunity costs are underway [10]. In the interim, preliminary analyses using estimates of foregone benefit from the English National Health Service and international income elasticities of the value of health have produced threshold values for a number of countries, including South Africa [10].

Here, we present the results of a recent modelling exercise undertaken for the South African government as part of the South African Investment Case which produced an alternative way of approximating the government’s willingness to pay for one specific set of interventions, HIV services [11]. The South African HIV Investment Case was commissioned by the South African Department of Health and the National AIDS Council in 2015 and aimed at identifying the optimal mix of HIV services under a constrained budget [11]. Funding for the public-sector HIV programme is the largest health budget item in South Africa, largely funded by the South African government with modest but important contributions from the US President’s Emergency Plan for AIDS Relief (PEPFAR) and the Global Fund to Fight AIDS, TB and Malaria (GFATM) [11]. Expenditure on HIV has increased by 19% on average year-on-year in real terms since the start of the country’s ART programme in 2004, and has outpaced the growth in the health budget overall in recent years [12]. The results of the HIV Investment Case were used to inform future HIV policy and guidelines and justify continued funding by the three funders mentioned above. Using the relationship between the currently committed HIV budget from the three main funders and modelled estimates of life years saved by a number of HIV prevention and treatment interventions, we were able to derive a revealed WTP threshold per life year saved, which we can then compare to the existing thresholds based on GDP per capita or opportunity cost.

## Methods

Using Thembisa/Optimise, a novel optimization method in association with Thembisa, an established model of the South African HIV epidemic, we developed a league table of HIV options (defined as the combination of an intervention and one of seven coverage levels) ordered by their cost-effectiveness ratio (ICER) over a rolling baseline [11]. After a comprehensive review process that has been described in detail elsewhere [11], we included 16 HIV prevention, testing and treatment interventions with proven effectiveness. Intervention coverage was estimated for the baseline year based on government reports. We then examined the impact of scaling each intervention up or down to up to 6 coverage levels, including the baseline coverage level, up to 2 equidistant coverage levels below baseline, the feasible maximum coverage level, and up to 3 equidistant coverage levels between baseline and the maximum. This resulted in 101 distinct intervention-coverage options. The feasible maximum represented an upper bound on the coverage level that could be reached by 2018/19 and was set at 70% for novel interventions and 95% for most existing interventions, except for male medical circumcision and HIV counselling and testing of the general population which were set to the model’s demand assumptions and based on government data, respectively. For each intervention that was present at baseline, we tested both increases and decreases in coverage while novel interventions were solely scaled up.

Cost effectiveness was expressed as incremental cost across the entire HIV programme per life year saved over 20 years (2016–2035) [13]. We estimated the epidemiological impact in terms of life years saved relative to the West level 26 life table, a life table representing population survival at very low levels of HIV prevalence [14]. The cost of each intervention was estimated from the government’s perspective and based either on our own cost analyses, literature with relevance to South Africa that was updated to the most recent input prices, or, in a few cases, expenditure data of relevant service providers. Cost was treated as uniform and presented unadjusted for inflation in 2016 USD (i.e. real 2016 USD). Neither cost nor outcomes were discounted (i.e. a 0% real discount rate).

The optimization method has been described in detail elsewhere [13]. It used a rolling baseline that started with the most cost-effective intervention (lowest ICER) and then sequentially added interventions in order of incremental cost-effectiveness, with the baseline re-estimated after the addition of each new intervention. In contrast to an analysis based on standard league table methodology, our approach allowed us to take into account the diminishing marginal returns of each added intervention, thereby decreasing each additional intervention’s cost effectiveness [13]. By considering South Africa’s entire HIV response and the dynamics of the epidemic, the model took into account the impact of HIV prevention services on reducing the need for treatment, and the impact of treatment on reducing transmission [13], which reduces the need for future prevention and treatment.

After the addition of each option, we compared the total cost of the package of services to the budget for HIV from three main funders—the South African Government, PEPFAR, and GFATM—for the years 2016–2018, the only years for which funds had been committed under the planning horizons of the three funders. We constrained the list of interventions to those whose combined cost was below the three funders’ combined annual HIV budget of $1.64 billion in 2016, $1.62 billion in 2017, and $1.69 billion in 2018 [11,13], resulting in a list of interventions ordered by cost effectiveness over 20 years that was also affordable in the short term. We allowed annual costs to increase as needed beyond the first 3 years for which budget data was available.

## Results

We report the incremental cost and life years saved as well as the ICER of each intervention option in Table 2, with the ICER calculated as the additional cost of the intervention divided by the additional life years saved. For each intervention, the highest possible coverage level was chosen by the optimization coverage. The top section includes those interventions that are affordable under the current budget. The bottom section lists the interventions whose total cost, in addition to the package in the top section, would surpass the current budget. Of note is that two interventions were found to be cost-saving from year one onwards—maximizing condom availability and use and maximizing male medical circumcision—due to their impact on incidence and, hence, treatment need.

**Table 2:** *Results of HIV investment case optimization over 20 years*, based on [13]. The thick black line marks where the willingness-to-pay threshold falls in relationship to the total cost of the package of interventions.

Intervention	Number of people in target population	Cost for target population	Total cost of HIV programme	Incremental cost (2016 USD)	Life years saved across HIV programme	ICER (USD/LYS)
*Affordable under current budget (2016–2018)*:						
Condom availability	820,091,500	1,031,393,274	38,676,468,274	-1,186,726,042	3,965,673	Cost saving
Male medical circumcision (MMC)	7,230,316	721,518,982	38,666,604,943	-9,863,330	965,043	Cost saving
ART under previous guidelines^1^	108,828,216	27,555,721,520	38,846,265,756	172,896,839	2,064,841	84
Prevention of mother-to-child transmission^2^	181,923	868,004	38,904,457,700	58,191,944	566,170	103
Universal treatment	3,496,802	90,007,293.31	38,934,004,197	29,546,497	157,938	187
Infant testing at 6 weeks	113,474,472	28,711,097,364.64	39,949,581,354	1,015,577,157	5,277,221	192
SBCC campaign 1 (HCT, reduction in partners)^3^	184,138,286	125,583,852.02	40,335,495,183	50,160,675	91,660	547
*Not affordable under current budget*:						
SBCC campaign 2 (condoms)^3^	976,957,287	340,640,616	40,807,306,076	16,893,385	19,377	872
General population HCT	821,714,255	2,552,842,627	41,028,577,881	222,973,294	239,361	932
SBCC campaign 3 (condoms, HCT, MMC)^3^	1,079,270,229	206,220,393	41,158,426,991	191,245,814	139,266	1,373
HCT for sex workers	2,367,552	22,162,261	41,189,826,427	31,399,437	15,674	2,003
Infant testing at birth	14,941,943	405,839,427	41,605,455,421	100,935,445	45,750	2,206
PrEP for sex workers	1,131,508	205,981,140	41,761,128,193	155,672,772	20,831	7,473
HCT for adolescents	98,369,912	2,070,189,640	44,020,229,887	498,523,420	32,581	15,301
PrEP for young women	55,862,810	9,987,554,706	52,261,161,471	8,240,931,585	412,361	19,985
Early infant male circumcision	7,702,676	325,027,144	52,534,654,763	206,823,176	3	68,941,059

USD, US dollars; ICER, incremental cost-effectiveness ratio;; ART, antiretroviral treatment; SBCC, social and behaviour change communication; HCT, HIV counselling and testing; PrEP, pre-exposure prophylaxis. Note that all numbers are summed over 20 years (2016–2035).

^1^Eligibility at 500 CD4 cells/microl.

^2^Initiation of triple ARV therapy for pregnant women not yet on lifelong ART.

^3^For the SBCC campaigns, the text in brackets summarises the main behavior change message(s).

The current HIV budget of about $1.6 billion per year was sufficient to pay for the expansion of condom availability, medical male circumcision, universal treatment, and infant testing at 6 weeks to maximum coverage levels, while also implementing a social and behavior change mass media campaign with a message geared at increasing testing uptake and reducing the number of sexual partners. The combined ICER of this package of services was $547/LYS. The ICER of the next intervention that was below the affordability threshold was $872/LYS.

Even though it aimed to identify the optimal mix of services against HIV under a constrained budget, the HIV Investment Case also produced data that shed light on the South African government’s willingness to pay for a unit of benefit from HIV services, which can then be compared to existing thresholds. This comparison is shown in Table 3.

**Table 3:** Comparison of cost-effectiveness thresholds.

Threshold	Unit	ICER
Our analysis (lower limit)	$/Life year saved	$547 [11]
Our analysis (upper limit)	$/Life year saved	$872 [11]
GDP per capita 2016	$/DALY averted	$6,000 (based on [2])
Opportunity cost (lower limit, preliminary estimate)	$PPP/DALY averted	$1,175 [10]
Opportunity cost (upper limit, preliminary estimate)	$PPP/DALY averted	$4,714 [10]

ICER, incremental cost-effectiveness ratio; GDP, gross domestic product; DALY, disability-adjusted life year. Note that all values presented here are unadjusted for purchasing power, while the WHO recommends using the adjusted GDP per capita value as a threshold (which would increase the value).

As shown in Table 3, current GDP per capita in South Africa (about $6,111 in 2016 [15]) is approximately 11 times higher than the ICER we estimated (the lower value; or 7 times our upper value). Preliminary estimates using the PPP-adjusted values from the opportunity cost method suggest a threshold that is 3 to 10 times the upper value we calculated. We note that converting life years gained to DALYs avoided would have the effect of reducing the ICERS we estimated, as using DALYs would add years of life lived in suboptimal health to our numbers of life years lost, thereby increasing the denominator of the ICER.

## Discussion

Using data from a recent, comprehensive analysis of the cost-effectiveness of HIV interventions in South Africa, we found that the country’s demonstrated willingness to pay, as revealed by its current budget, is far less than suggested by the international cost-effectiveness thresholds most often used. The results of the South African HIV Investment Case point to a cost-effectiveness threshold for HIV services based on affordability under the current budget of $547–872 per life year saved, a small fraction of the country’s GDP per capita or the estimated opportunity cost of healthcare per DALY averted. These results can help place standard cost-effectiveness analyses in context, by demonstrating how close to “affordable” an intervention that is labeled cost-effective based on GDP/capita is for the country in question. As indicated in Table 3, South Africa’s GDP/capita is roughly $6000, making an intervention highly cost effective if it costs less than $6000/LYS. The last affordable intervention in our league table, however, suggests that affordability may be closer to $500/LYS, providing readers with some perspective on the feasibility of adopting even an intervention that appears “highly cost effective”. In addition to reporting an ICER, coverage levels and the annual additional budget needed to cover the intervention would be part of the presentation of results.

For practical purposes, using thresholds based on GDP per capita or opportunity costs, while useful in allowing for cross-national comparisons and serving as aspirational goals, does not provide the South Africa Government with relevant information for determining whether implementing proposed new interventions, or increases in coverage with existing interventions, is affordable. Our league table approach avoids these concerns. As the number of interventions included depends on the available budget, a league table that addresses affordability along with cost-effectiveness. The last intervention accepted on a league table is therefore a closer estimation of a government’s willingness to pay than standard thresholds [4]. If the overall budget is held constant, however, any new interventions would need to be at least as cost-effective as the package of care that defines the threshold (i.e. have an ICER below our calculated limit) *and* be affordable under the current budget, possibly by replacing the next less cost-effective intervention. This depends, in turn, on fungibility of resources across diseases, interventions, and programmes, a condition that is constrained by targeted donor funding in many countries. However, we believe that our results can be used to also make the case for non-HIV interventions that save more life years at < $500 than those HIV interventions that we have included.

In interpreting this finding, it is important to consider the limitations of our analysis. First, we included the committed budget from three funders, including PEPFAR and GFATM, not just the South African government. PEPFAR and GFATM funds were earmarked for HIV programmes and could not be used for other purposes. However, if we had included funding from the South African government only, we would have likely excluded the specific interventions that make up these programmes as well, so the overall impact on the optimal package or its marginal ICER would have been small. Furthermore, in a situation in which the South African government contributed 85% of the budget [11] and had a certain flexibility in using these funds for other purposes, however, we believe that the threshold we calculated is a reasonable representation of the government’s willingness to pay. Second, we recognize that revealed WTP based on existing government and donor budgets does not necessarily comprise a society’s full WTP. Patients themselves may be willing to pay privately to gain access to healthcare interventions beyond the current government budget. Governments may also be willing to increase its budget allocation if the value of a more expensive intervention is demonstrated. Third, we are aware that our use of a rolling baseline results in lower ICERs across interventions than in CEAs using standard methodology which does not account of diminishing marginal returns [13]. In a separate review of published cost and cost effectiveness analyses of HIV interventions in South Africa that were based on primary data, we found ICERs for ART between of $1,2334 per life year gained [16] to $8,865 per extra patient retained in care after 10 years [17] to $23,444 per death averted [18], and ICERs for HIV counselling and testing of between $146 [19] and $21,920 [20] per infection averted (all in 2016 USD) [21]. We however believe that our method is more appropriate especially in the context of a mature HIV programme such as South Africa’s, in which most interventions are already delivered at a high coverage level at baseline [13]. Lastly, by constraining the optimal mix of interventions over 20 years by what could be afforded over the first 3 years, we might have chosen a sub-optimal investment strategy. However, the only budget data available were for those years. The current financial climate in particular in South Africa (which has entered a recession in 2017, after decades of growth) makes it hard, if not impossible, to project budgets over the mid-term, and we did find that even under the constrained optimization scenario budgets will have to grow by more than current inflation year-on-year for the foreseeable future.

We believe that our findings can contribute to the discussion about cost-effectiveness thresholds more generally. As noted in the Introduction, the recommendation for GDP-based thresholds has recently been weakened, as these thresholds have been found to be not a good proxy for utility [4], not a good decision tool for middle-income countries such as South Africa, as the majority of health interventions will have ICERs that pass below [3], and ignorant of the opportunity costs of healthcare spending [8]. The body that originally recommended these thresholds, the WHO CHOICE team [2], has since withdrawn this recommendation [5,22]. Opportunity-cost based thresholds have been suggested as an alternative, with work on the relationship between income and the value of a statistical life based on the UK National Health Service [8] extrapolated to a number of other countries, assuming a similar relationship [10], while more local estimations are ongoing, including for South Africa [23].

We conclude that while the WTP threshold found in the South African HIV Investment Case should by no means be used as the sole criterion for determining the cost effectiveness or desirability of HIV services in South Africa, it does indicate the large gulf that separates actual WTP from existing thresholds based on income or opportunity cost. It also defines a “benchmark” threshold that can assist policy makers in understanding how a proposed new programme compares to what they are already doing [4]. This analysis thus allows us to incorporate revealed preferences into the ongoing debate on how to understand and utilize cost-effectiveness estimates for healthcare decision making in resource constrained settings.
